# Chemokine CCL1 as a therapeutic target for pulmonary fibrosis: *comments on* ‘*The chemokine CCL1 triggers an AMFR‒SPRY1 pathway that promotes differentiation of lung fibroblasts into myofibroblasts and drives pulmonary fibrosis*’

**DOI:** 10.1093/jmcb/mjab080

**Published:** 2021-12-23

**Authors:** Shanshan Liu, Chang Liu, Zhuowei Hu, Yang Xiao

**Affiliations:** National Clinical Research Center for Metabolic Diseases, Key Laboratory of Diabetes Immunology, Ministry of Education, and Department of Metabolism and Endocrinology, The Second Xiangya Hospital of Central South University, Changsha 410011, Hunan, China; Drug Clinical Trial Institution, Children’s Hospital, Capital Institute of Pediatrics, Beijing 100020, China; National Clinical Research Center for Metabolic Diseases, Key Laboratory of Diabetes Immunology, Ministry of Education, and Department of Metabolism and Endocrinology, The Second Xiangya Hospital of Central South University, Changsha 410011, Hunan, China; National Clinical Research Center for Metabolic Diseases, Key Laboratory of Diabetes Immunology, Ministry of Education, and Department of Metabolism and Endocrinology, The Second Xiangya Hospital of Central South University, Changsha 410011, Hunan, China

Pulmonary fibrosis (PF) is a type of chronic and progressive respiratory diseases characterized by excessive extracellular matrix (ECM) deposition, interstitial fibrotic lesions, and architectural distortion. Patients with PF suffer from pulmonary function decline and progressive worsening of dyspnea with poor prognosis (Wilson and Wynn, 2009). Although recent progress provides mechanistic insights into the pathogenesis of PF, no effective treatment against PF is available other than lung transplantation. Therefore, a better understanding of the molecular and cellular mechanisms of PF is crucial for the discovery of new therapeutic targets for safe and effective anti-PF drugs.

Chemokines (chemotactic cytokines, 8‒10 kDa) are a family of small soluble proteins that were originally discovered as mediators of the directional migration of immune cells such as monocytes, natural killer (NK) cells, immature B cells, and dendritic cells to the sites of inflammation and injury (Lau et al., 2020). Accumulating evidence suggests that chemokines are involved in the pathogenesis of PF (Prasse et al., 2006; Milger et al., 2017) through mediating the infiltration of immune cells into the injured lung or directly activating fibroblasts (the effector cells in PF). Here, chemokine (C–C motif) ligand 1 (CCL1), also known as I-309 in humans and TCA-3 in mice, is a small glycoprotein secreted by activated T lymphocytes. By interacting with CCR8, a cell-surface chemokine receptor, CCL1 physiologically functions as a mediator of the directional migration of immune cells to the sites of inflammation and injury (Gombert et al., 2005; Knipfer et al., 2019). Pathologically, CCL1 is involved in a variety of chronic pulmonary diseases, including chronic obstructive pulmonary disease, bronchial asthma, and lung adenocarcinoma. However, the exact roles of chemokines, especially CCL1, in the pathogenesis of PF are still unclear.

**Figure 1:**
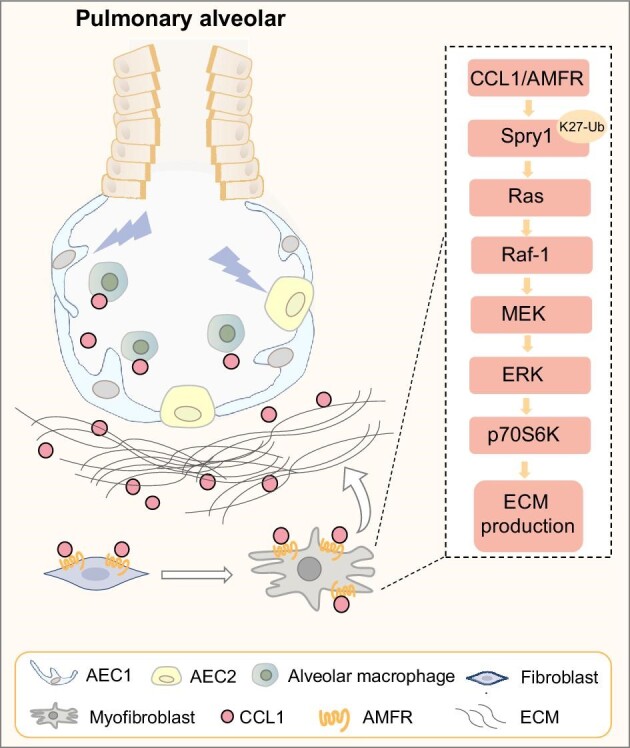
Illustration of the CCL1 working model in pulmonary fibrosis. Chronic lung injury triggers the release of CCL1 from activated alveolar macrophages, and this in turn activates lung fibroblasts by binding to AMFR on the membrane of these cells, thereby driving fibrogenic protein synthesis by activating the Ras‒ERK‒p70S6K pathway. AEC1, type I alveolar epithelial cells; AEC2, type 2 alveolar epithelial cells.

Liu et al. (2021) found that the expression of CCL1 is upregulated in lung tissues of PF patients and mice. CCL1 exerts its fibrogenic activity by signaling through autocrine motility factor receptor (AMFR) on lung fibroblasts and activating those cells into pathological myofibroblasts. Mechanistically, elevated CCL1 causes protein kinase C alpha-mediated phosphorylation of AMFR, through which AMFR acquires E3 ligase activity that enables it to ubiquitinate and translocate the endogenous extracellular signal-regulated kinase (ERK) inhibitor sprouty RTK signaling antagonist 1 (Spry1) to the plasma membrane. On the plasma membrane, Spry1 binds to Ras GTPase activating protein, thereby relieving the inhibitory effect of Spry1 on the Ras‒ERK‒p70S6K signaling activity and enhancing profibrotic protein synthesis in fibroblasts. Genetic and pharmacological inhibition of the CCL1 signaling pathway exhibits potent therapeutic efficacy against PF (Figure 1).

Previous studies have shown that CCR8 acts as the only known receptor for CCL1. However, CCL1 is able to effectively activate CCR8-depleted fibroblasts to the same extent as CCR8-positive fibroblasts, indicating an additional unknown CCL1 receptor that mediates fibroblast activation. By using mass spectrometry analysis, AMFR is identified as a novel CCL1 receptor that activates fibroblasts. AMFR, a RING finger-dependent E3 ligase, is reported to catalyze K48- or K27-linked polyubiquitination of its substrates (Wang et al., 2014; Menzies et al., 2018). Here, in response to CCL1, AMFR acquires its E3 ligase activity and induces the K27-linked ubiquitination of Spry1. AMFR acts as an E3 ubiquitin ligase only when it is localized to the smooth endoplasmic reticulum membrane (Chen et al., 2006). In this study, unlike the previous reports, the authors revealed that AMFR can also exhibit its E3 ligase activity when it is localized on the cell membranes. Taken together, for the first time, the study by Liu et al. (2021) identifies AMFR as another natural receptor for CCL1 to exert biologic effects.

As with any novel finding, several questions remain unanswered. Because CCR8 and AMFR are also expressed on alveolar macrophages and T cells, which contribute to the pathogenesis of PF, the underlying molecular mechanisms of CCL1 on those AMFR^+^ or CCR8^+^ cells need to be determined. Given the biological significance of CCL1 in a broad range of human chronic pulmonary diseases, the CCL1‒AMFR signal cascade is speculated to contribute to the progression of these diseases. To explore this notion, investigators from the Second Xiangya Hospital of Central South University have recently started to use AMFR and/or CCR8 knockout mice to examine the exact pathogenesis roles of CCL1 in these CCL1-associated pulmonary disorders. These ongoing studies in the Second Xiangya Hospital team will help us understand the in-depth mechanisms of CCL1 and its receptors in cell or disease context. Moreover, toward figuring out their respective mechanisms, small-molecule screens will be carried out to specifically interfere with the CCL1‒CCR8 or CCL1‒AMFR function.

Overall, the study by Liu et al. (2021) elucidates the crucial roles played by CCL1 and its novel receptor AMFR in the pathogenesis of PF, presenting the CCL1‒AMFR‒ERK signal cascade as a promising target for therapeutic intervention. This work not only expands our knowledge of CCL1 biology, but also sheds new light on therapeutic strategies for curing fibroproliferative lung diseases.

*[The work described was supported by grants from the National Key R&D Program of China (2018YFE0114500), the ‘361 Project’ Outstanding Young Talent of the Second Xiangya Hospital of Central South University, the National Natural Science Foundation of China (81803604), and the National Science Foundation of Hunan Province for Excellent Young Scholars (2020JJ3056).]*
